# GABA potentiate the immunoregulatory effects of *Lactobacillus brevis* BGZLS10-17 via ATG5-dependent autophagy *in vitro*

**DOI:** 10.1038/s41598-020-58177-2

**Published:** 2020-01-28

**Authors:** Svetlana Soković Bajić, Jelena Đokić, Miroslav Dinić, Sergej Tomić, Nikola Popović, Emilija Brdarić, Nataša Golić, Maja Tolinački

**Affiliations:** 1 Laboratory for Molecular Microbiology (LMM), Institute of Molecular Genetics and Genetic Engineering (IMGGI), University of Belgrade, Belgrade, Serbia; 2 Department for Immunology and Immunoparasitology, Institute for the Application of Nuclear Energy, University of Belgrade, Belgrade, Serbia

**Keywords:** Immunosuppression, Applied microbiology

## Abstract

The characterization of mechanisms involved in the positive effects of probiotic bacteria in various pathophysiological conditions is a prerogative for their safe and efficient application in biomedicine. We have investigated the immunological effects of live bacteria-free supernatant collected from GABA-producing *Lactobacillus brevis* BGZLS10-17 on Concanavalin A-stimulated mesenteric lymph node cells (MLNC), an *in vitro* model of activated immune cells. We have shown that GABA containing and GABA-free supernatant of *Lactobacillus brevis* BGZLS10-17 have strong immunoregulatory effects on MLNC. Further, GABA produced by this strain exhibit additional inhibitory effects on proliferation, IFN-γ and IL-17 production by MLNC, and the expression of MHCII and CD80 on antigen presenting cells. At the other hand, GABA-containing supernatants displayed the strongest stimulatory effects on the expression of immunoregulatory molecules, such as Foxp3^+^, IL-10, TGF-β, CTLA4 and SIRP-α. By looking for the mechanisms of actions, we found that supernatants produced by BGZLS10-17 induce autophagy in different MLNC, such as CD4^+^ and CD8^+^ T lymphocytes, NK and NKT cells, as well as antigen presenting cells. Further, we showed that the stimulation of Foxp3^+^, IL-10 and TGF-β expression by BGZLS10-17 produced GABA is completely mediated by the induction of ATG5 dependent autophagy, and that other molecules in the supernatants display GABA-, ATG5-, Foxp3^+^-, IL-10- and TGF-β- independent, immunoregulatory effects.

## Introduction

The incidence of exacerbated immune response-related diseases, especially autoimmune diseases, has been rising steadily^1^ but the new approaches for their treatment have not been developed sufficiently. The discovery on the important role of gut microbiota in maintaining host’s immune homeostasis^2^ initiated the research on immunomodulatory potential of microorganisms. In addition, to decipher the role of commensal members of gut, probiotic bacteria with potentially beneficial effects on the hosts are isolated from different sources. Probiotic bacteria are defined as ‘live microorganisms that, when administered in adequate amounts, confer a health benefit on the host’^3^. Additionally, live bacteria-free fractions of probiotics (postbiotics) could provide beneficial effects without the potential risk associated with the administration of live microorganisms^4^. Besides being defined as beneficial to health, the best and the safest way to apply these bacteria and/or their products in clinics is to determine the specific mechanisms of probiotic activity in appropriate immune model systems. In that sense, the mechanisms of immunomodulatory activity of different probiotic strains should be fully characterized, thus enabling the development of specific treatment for particular immune-related disease. Some of the bacterial active molecules are also being produced by eukaryotes, so the supplementation of host with such probiotic bacteria, or their postbiotic preparation, is a natural way to modulate the host’s immune functions. One of such molecules is γ-aminobutyric acid (GABA), a well-recognized for its role as the main inhibitory neurotransmitter^5^. In addition, GABA receptors have been identified in a wide range of immune cells, and they are involved in a general down-regulation of proinflammatory cytokines production, making GABA an important immunomodulator as well^6^. The immunomodulatory role of chemically synthesized GABA has been observed in autoimmune diseases such as mouse model of rheumatoid arthritis^7^, mouse model of obesity^8^, type 1 diabetes^9^, and experimental autoimmune encephalomyelitis (EAE)^6^, suggesting that GABA could be applied as an adjuvant treatment against autoimmune and inflammatory diseases. In addition to synthetic, there are simple biosynthetic procedures of GABA with high efficiency and environmental compatibility^10^. The promising source of GABA for the potential treatment of exacerbated inflammation are probiotic bacteria, considering the fact that main producers of GABA belong to lactic acid bacteria (LAB)^11^ which have the “Qualified Presumption of Safety” (QPS) status^12,13^.

One of the important mechanisms of immune response regulation is autophagy^14^. Next to the pivotal role of autophagy in eradication of intracellular pathogens, autophagy is a homeostatic mechanism primarily involved in the removal of damaged organelles and denatured proteins through a lysosomal degradation pathway^15^. The autophagy proteins act in both the induction and the suppression of immune responses and vice versa, the inflammatory signals function as inducers and/or suppressants of autophagy^16^. Importantly, literature data suggest that deregulation of autophagy is associated with various conditions characterized by exacerbated inflammation^17^. Interestingly, the activation of GABAergic signaling is positively implicated in the treatment of these conditions, but based to our knowledge, the potential relation of autophagy and GABAergic signaling in inflammatory conditions have not been investigated so far. Besides the role of GABA in the induction of antimicrobial autophagy^18^, our group as well as studies from other groups reported the potential of some *Lactobacillus* species to stimulate or suppress autophagy^19–21^. In addition, the role of autophagy in immunomodulatory effects of GABA-producing probiotic strain have not been investigated. We have recently reported on the protective effects of GABA-producing *Lactobacillus brevis* BGZLS10-17, a natural isolate from artisanal Zlatar cheese^22^. In this system *L. brevis* produced GABA only in the presence of monosodium glutamate (MSG) and displayed protective effects in an *in vitro* model of inflammation-induced destruction of intestinal barrier. Interestingly, it was shown that L-glutamate, a precursor of GABA, could be produced from dairy proteins by some bacteria (*Streptococcus thermophilus*) enabling the production of GABA-rich products by GABA-producing bacteria^23^ Therefore, in this study we investigated further whether this GABA-producing strain exhibit immunomodulatory effects via soluble route. Recent work of Engevik *et al*.^24^, demonstrated that GABA and other soluble molecules produced by *Bifidobacterium dentium* stimulate the expression of molecules involved in epithelial barrier function by autophagy-dependent mechanisms. These authors assumed that this mechanism could contribute to protective roles of this bacterium in inflammatory conditions. Besides GABA, other soluble molecules with immunomodulatory effects were described to be produced by LAB, and these are being investigated as potentially useful postbiotics^25^. However, the relation between GABA-producing LAB and their immunomodulatory properties has not been investigated previously. Considering recent findings on potential linkage of GABA signaling and autophagy^18^, we hypothesized that the direct immunomodulatory activity of GABA produced by *L. brevis* BGZLS10-17 include the regulation of autophagy within these immune cells.

## Results and Discussion

### Supernatants from *Lactobacillus brevis* BGZLS10-17 have immunoregulatory effects at non-toxic doses

The emerging new evidences suggests that the GABA signalling is involved in maintenance of immune system homeostasis^26^. Therefore, the ability of probiotic strains to produce GABA seems as a good strategy to modulate immunological responses in different inflammatory diseases. In our previous study, we tested the GABA-producing ability of different LAB strains isolated from dairy products, and found that *L. brevis* BGZLS10-17 displays the strongest capacity to produce GABA. Namely, this live bacteria-free GABA-containing supernatant (4 mM GABA in 2.5% supernatant; MRS/MSG) inhibited the inflammation induced-destruction of gut epithelial cell barrier significantly more than the corresponding supernatants which did not contain GABA (MRS)^22^. Moreover, by using an *in vivo* model of inflammatory disease, i.e. experimental autoimmune encephalomyelitis (EAE) (an animal model of multiple sclerosis), we found that the oral administration of live bacteria or 48 h bacteria-free supernatant containing GABA, alleviated the EAE symptoms in this model^27^, pointing to their immunoregulatory effects *in vivo*. To further elucidate the mechanisms of *L. brevis* BGZLS10-17 actions, our aim in this work was to investigate the immunomodulatory effects of GABA-producing *L. brevis* BGZLS10-17 strain by using a model of Concanavaline A (ConA)-stimulated mesenteric lymph node cells (MLNC), as MLN is a critical secondary lymphoid organ draining guts. Con-A is commonly used as a polyclonal activator of lymphocytes, crosslinking the molecules on antigen presenting cells and lymphocytes, which is followed by the stimulation of lymphocytes’ proliferation and cytokines production^28^. Therefore, in order to decipher the role of GABA produced by this strain, we tested and compared the effects of supernatants collected after the cultivation of *L. brevis* BGZLS10-17 in conditions where they do not produce GABA (MRS) and in GABA-producing conditions (in the presence of MSG-MRS/MSG). Additionally, the effects of GABA produced by *L. brevis* BGZLS10-17 during the cultivation were compared with the effects of artificial GABA added in the same concentration as found in the supernatant collected from bacterial culture without MSG (MRS/art.GABA).

**Figure 1:**
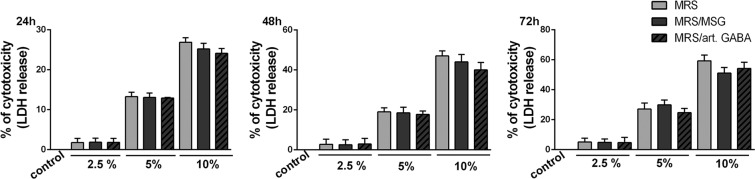
Dose-dependent toxicity in the culture of MLNC. Dose (2.5%, 5%, 10%) dependent effect of BGZLS10-17’s supernatants on ConA stimulated MLNC viability after 24, 48 and 72 h of treatment assessed by LDH assay. All values are presented as mean ± SD from three independent experiments; MRS-supernatant without GABA; MRS/MSG-supernatant with produced bacterial GABA; MRS/art. GABA-supernatant with the addition of artificial GABA.

**Figure 2:**
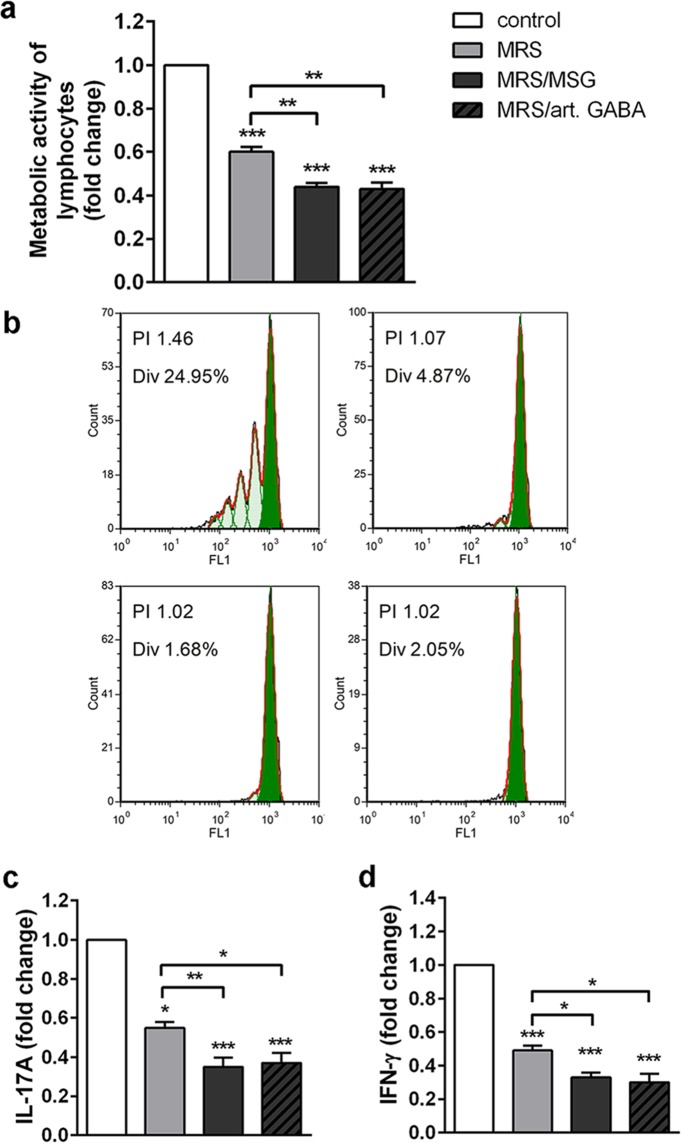
Immunoregulatory effects of BGZLS10-17 supernatants on MLNC. The effect of BGZLS10-17 supernatants on metabolic activity (MTT) (**a**), proliferation (**b**), and production of proinflammatory cytokines IL-17A (**c**) and IFN-γ (**d**) of ConA stimulated MLNC, after the 72 h treatment with 2.5% supernatant without GABA (MRS), 2.5% supernatant with 4 mM bacterial GABA (MRS/MSG), and 2.5% supernatant with addition of 4 mM artificial GABA (MRS/art. GABA). All values are presented as mean ± SD from three independent experiments. One-way ANOVA with the Dunnett’s test was used to compare multiple groups. The statistical significance of MTT and cytokine production is shown (*p < 0.05; **p < 0.01, ***p < 0.001).

In order to exclude the possibility that the immunological effects are due to cytotoxicity of the supernatants, we first analysed the dose dependent toxicity in the culture of MLNC. We found that 2.5% supernatants had no significant cytotoxic effects on stimulated MLNC after 24 h, 48 h and 72 h treatment (Fig. 1), unlike the higher doses (5% or 10%). The toxicity of supernatants at higher doses most likely comes from MRS medium components because there is no difference between treatments (MRS/MSG, MRS/art. GABA) and the control group (MRS). This is in accordance with the results published by other groups^29^. However, the treatments with non-cytotoxic concentrations of the supernatants (2.5%) significantly reduced the metabolic activity of ConA-stimulated MLNC, as compared to non-treated cells (Fig. 2a), pointing to the potential immunomodulatory effects of BGZLS10-17 supernatants. Interestingly, the cultures of ConA-stimulated MLNC treated with the supernatants containing GABA produced by *L. brevis* BGZLS10-17 strain or artificial GABA displayed significantly lower metabolic activity in comparison to the MLNC cultures treated with supernatant without GABA (MRS), which pointed to additional immunoregulatory effects of GABA contained in *L. brevis* BGZLS10-17 supernatants. The inhibitory effects of GABA-free *L. brevis* BGZLS10-17 supernatant could be mediated by other soluble molecules previously described to modulate functions of immune system such as protein p40^30^, lactocepin^31^ and other uncharacterized soluble molecules^29^. Additionally, we checked the effect of fresh MRS added in the same concentration as the supernatants from bacterial cultures (2.5% (12.5 μl) MRS), and found that the fresh MRS media had no significant effects on the metabolic activity of MLNC (Supplementary Fig. 1a).

To further investigate the immunomodulatory effects of the supernatants, we measured the proliferation of ConA-stimulated MLNC and the levels of IL-17 and IFN-γ production after 72 h cultures. These results showed that GABA-containing and GABA-free *L. brevis* BGZLS10-17 supernatants display strong anti-proliferative effects on ConA-stimulated MLNC (Fig. 2b). In accordance with this, all tested supernatants showed inhibitory effects on the production of proinflammatory cytokines, IL-17A and IFN-γ by MLNC (Fig. 2c,d). Again, GABA containing supernatants (MRS/MSG and MRS/art. GABA) had an additional inhibitory effect on the production of IL-17A and IFN-γ, as compared to the effects of supernatant without GABA (MRS). Thereby, the fresh MRS medium had no significant effects on the production of IL-17 and IFN-γ (Supplementary Fig. 1b,c). IL-17 and IFN-γ are the hallmarks of T helper (Th)17 and Th1 cells, respectively, which are critically involved in proinflammatory responses and exacerbation of autoimmune diseases^32^. The main immunoregulatory mechanism involved in downregulation of Th17 and Th1 cell responses involve the production of anti-inflammatory cytokines (IL-10 and TGF-β) by Foxp3^+^ regulatory T cells (Treg)^33^.

**Figure 3:**
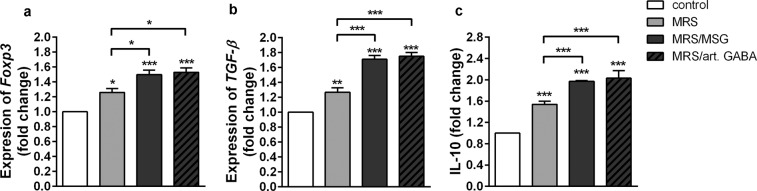
BGZLS10-17 supernatants increase tolerogenic properties of MLNC. The effect of BGZLS10-17 supernatants on mRNA level of *Foxp3* (**a**), mRNA level of *TGF-β* (**b**), and production of IL-10 (**c**) of ConA stimulated MLNC. MLNC were treated with 2.5% supernatant without GABA (MRS), 2.5% supernatant with 4 mM bacterial GABA (MRS/MSG), and 2.5% supernatant with addition of 4 mM artificial GABA (MRS/art. GABA). mRNA levels were measured after 24 h, while the production of IL-10 was measured after 72 h. All values are presented as mean ± SD from three independent experiments. One-way ANOVA with the Dunnett’s test was used to compare multiple groups. The statistical significance is shown (*p < 0.05; **p < 0.01, ***p < 0.001).

Therefore, we investigated how *L. brevis* BGZLS10-17 supernatants act on Foxp3, IL-10 and TGF-β expression in MLNC. We found that all tested supernatants significantly stimulated the expression of *Foxp3* mRNA and immunoregulatory cytokine *TGF-β* mRNA, as well as the production of anti-inflammatory cytokine IL-10 in comparison to non-treated ConA-stimulated MLNC. The higher levels of *Foxp3* mRNA and *TGF-β* mRNA were measured in MLNC treated with GABA containing supernatants (MRS/MSG and MRS/art. GABA) compared to supernatants without GABA (MRS) (Fig. 3a,b). In addition, GABA containing supernatants (MRS/MSG and MRS/art. GABA) displayed a stronger stimulatory effect on IL-10 production by MLNC (Fig. 3c).

These results indicated that *L. brevis* BGZLS10-17 supernatants exhibit immunoregulatory effects and induce Foxp3, IL-10 and TGF-β expression. However, they also suggest that GABA has an additional immunomodulatory effect in this model system. It was suggested previously that the autophagy have important roles in immune response homeostasis^16^, as well as in induction and functions of Foxp3^+^ Treg^34^. In accordance with that, we further analysed whether *L. brevis* BGZLS10-17 supernatants affect autophagy in MLNC.

### Supernatants from *Lactobacillus brevis* BGZLS10-17 induce autophagy in MLNC

The process of autophagy is commonly monitored by the expression analysis of different proteins associated with autophagosome initiation, nucleation, elongation and regulation^35^. The most indicative marker for autophagy is the conjugation of cytosolic form of microtubule-associated protein light chain 3, LC3 (LC3I), with a phosphatidylethanolamine (PE) that, when integrated into the autophagosomal membrane is named LC3II^36^. LC3II remains associated during the whole autophagy process, and the closed autophagic vesicle is then addressed to lysosomes during the maturation phase. Therefore, the expression of LC3I and II in Con-A-stimulated MLNC treated with *L. brevis* BGZLS10-17 was monitored after 24 h (Supplementary Fig. 2a). It was observed that only GABA-containing supernatants (MRS/MSG and MRS/art.GABA) significantly increased the ratio of LC3II/LC3I. Surprisingly, GABA-free (MRS) supernatants increased LC3II/LC3I ratio, but the effect was not statistically significant. Since the ratio of LC3II/LC3I might be affected by the simultaneous lysosomal degradation of LC3II, we also checked whether similar phenomenon could be observed with the blocking of lysosomal acidification by chloroquine (CQ) (Suplementary Fig. 2b). In these experiments, all supernatants significantly increased the LC3II/LC3I ratio compared to control. However, GABA-containing supernatants, again, displayed significantly stronger effects on the upregulation of LC3II/LC3I ratio compared to GABA-free (MRS) supernatant.

**Figure 4:**
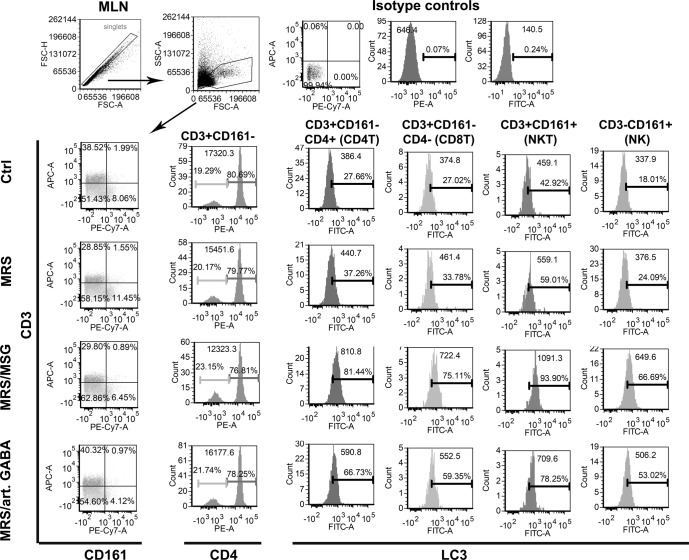
The influence of BGZLS10-17 supernatants on autophagy of ConA stimulated different types of T cells (CD4T, CD8T, NKT, and NK). The percentage and mean of CD3^+^CD161^−^CD4^+^LC3^+^, CD3^+^CD161^−^CD4^−^LC3^+^, CD3^+^CD161^+^LC3^+^, CD3^−^CD161^+^LC3^+^ cells were assessed by flow cytometry. MLNC were treated with 2.5% supernatant without GABA (MRS), 2.5% supernatant with 4 mM bacterial GABA (MRS/MSG), and 2.5% supernatant with addition of 4 mM artificial GABA (MRS/art. GABA) for 24 h. Representative histograms from two experiments are presented.

MLNC is a heterogeneous cell population containing different leucocytes including T, B, nature killer (NK), NK T cells (NKT), dendritic cells (DC) and macrophages (Mf). The proliferation and the cytokines production in the model system of ConA-induced stimulation of MLNC rely on lectin mediated crosslinking of surface receptors on antigen presenting cells (APC predominantly DC, Mf and B cells) and responder lymphocytes^28^. Considering that the increase of LC3II/LC3I ratio was detected in whole MLNC population, we next wondered which population of MLNC is specifically affected by *L. brevis* supernatants. Thereby, the LC3II expression was monitored after the intracellular staining^37^ of CQ treated MLNC, 24 h after the treatment with supernatants. The discrimination between T, NKT and NK cells was monitored according to CD3 and CD161 surface staining, as previously described^38^. It was found that the highest up-regulation of LC3II occurs within CD4^+^T, CD8^+^ T, NK (CD161^+^ CD3^−^) and NKT (CD161^+^ CD3^+^) cells (Fig. 4). Thereby, GABA-containing supernatants (MRS/MSG and MRS/art.GABA) showed the strongest effect on up-regulation of LC3II. Taking into account that within these cells lie the main producers of IFN-γ and IL-17^39,40^, the suppression of IL17 and IFN-γ by GABA containing supernatants could be explained by autophagy-dependent mechanisms. Also, the induction of autophagy in CD4^+^ T cells could be involved in the mechanism of Treg (CD4^+^ CD25^+^ FoxP3^+^) induction and their functions such as increased IL-10 and TGF-β production^41^. The production of anti-inflammatory cytokines IL-10 and TGF-β is not an exclusive feature of Tregs, but they could be produced by different tolerogenic immune cells, such as regulatory B cells, DC and Mf^42^. These cytokines were shown to prevent DC activation and induce the generation of tolerogenic DC as well^43–45^.

**Figure 5:**
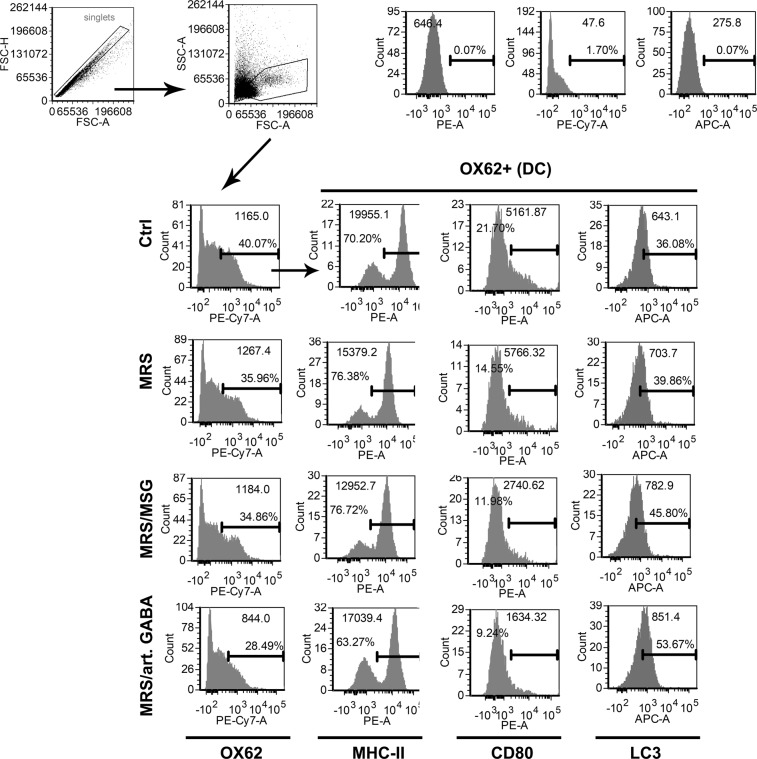
The influence of BGZLS10-17 supernatants on the expression of pro-inflammatory molecules MHC-II and CD80 and autophagy of dendritic cells (DC). The percentage of OX62^+^ MHC-II^+^, OX62^+^ CD80^+^, and OX62^+^ LC3^+^ was assessed by flow cytometry. MLNC were treated with 2.5% supernatant without GABA (MRS), 2.5% supernatant with 4 mM bacterial GABA (MRS/MSG), and 2.5% supernatant with addition of 4 mM artificial GABA (MRS/art. GABA) for 24 h. Representative histograms from two experiments are presented.

With that in mind, we also analysed the expression of LC3II within DC (OX62^+^) (Fig. 5), B cells (HIS24^+^ cells, Supplementary Fig. 3) and macrophages (CD68^+^ cells, Supplementary Fig. 4). The analysis showed that LC3 expression is indeed stimulated in all these cells by the supernatants, but the increase in APC was less pronounced than in T lymphocytes, NKT and NK cells. GABA-containing supernatants induced the expression of LC3II within all APC (OX62^+^DC, B cells and Mf). In contrast, GABA-free (MRS) supernatants induced LC3II expression in DC and Mf, but not in B cells. According to the analysis of MHC class II expression, the supernatants did not affect significantly% of MHC class II^+^ DC, Mf and B, but the relative expression/cell (MFI) was reduced after the treatment with supernatants. Autophagy was shown previously to be tightly regulated with MHC class II expression and antigen presentation^46^, but we also described that it is involved in immunomodulation and induction of tolerogenic DC^47^. In line with this, here we found that DC and B cells displayed lower expression of CD80 upon the treatment with supernatants, especially with GABA-containing supernatants. Additionally, we observed that the expression of checkpoint inhibitor CTLA-4 on B cells is up-regulated after the treatment with *L. brevis* supernatants. CD80 was shown to deliver stimulatory signal to T cells via CD28^48^. However, CD80 can also interact with CTLA-4, which rather provide immunoregulatory signals and induces regulatory T cells and regulatory B cells^49^. Additionally, it was shown that CTLA-4 expression, similarly as Foxp3, is under the control of autophagy^50^. Therefore, both down-regulation of CD80 and up-regulation of checkpoint inhibitor CTLA-4 can lead to overall immunoregulatory effects observed in MLNC after the treatment with *L. brevis* supernatants. Besides CTLA-4, we observed that Mf treated with the supernatants also up-regulated another checkpoint inhibitor, SIRP-α. It was shown previously that CD47 ligation of SIRP-α on Mf, prevents their phagocytotic capacity and the induction of antitumor proinflammatory response, which was proposed as the basis for new checkpoint blockade system in cancer immunotherapy^51^. Besides, it was shown that this blockade could be potentiated by blocking autophagy simultaneously^52^, suggesting that the expression and functions of SIRP-α in Mf are positively regulated by autophagy.

### Supernatants from *Lactobacillus brevis* BGZLS10-17 induce different autophagy signaling depending on GABA presence in the supernatant

The roles of proteins involved in different autophagy dependent homeostatic or pathophysiological conditions are increasingly being proven. Autophagy-related gene (ATG) proteins are known as the ‘core machinery’ of autophagy^53^. Thus, Unc51-like kinase 1 (ULK1) complex initiates phagophore formation^54^. In the following sequences, ULK1 activates Beclin-1, which is implicated in the endoplasmic reticulum-derived phagophore formation^55^. Beclin-1 interacts with UV resistance-associated gene (UVRAG) to promote autophagosome formation^56^. When phagophore is formed, ATG5 is implicated in autophagosome elongation phase leading to the conversion of soluble cytosolic form of LC3 (LC3I) into lipid bound LC3II form. LC3 and GABARAP (gamma-aminobutyric acid A receptor) are two of seven members of the most studied autophagy related protein family LC3/GABARAP commonly presumed to have similar functions, but there are evidence pointing to unique role of different proteins in this family in autophagy-dependent as well as autophagy-independent mechanisms^57^.

**Figure 6:**
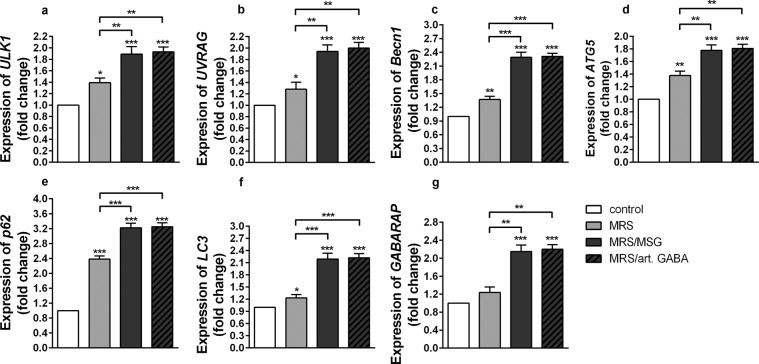
The influence of BGZLS10-17 supernatants on autophagy in MLNC. The effect of BGZLS10-17 supernatants on mRNA levels of *ULK1* (**a**), *UVRAG* (**b**), *Becn1* (**c**), *ATG5* (**d**), *p62* (**e**), *LC3* (**f**), and *GABARAP* (**g**) of ConA stimulated MLNC, after 24 h treatment. MLNC were treated with 2.5% supernatant without GABA (MRS), 2.5% supernatant with 4 mM bacterial GABA (MRS/MSG), and 2.5% supernatant with addition of 4 mM artificial GABA (MRS/art. GABA). All values are presented as mean ± SD from three independent experiments. One-way ANOVA with the Dunnett’s test was used to compare multiple groups. The statistical significance is shown (*p < 0.05; **p < 0.01, ***p < 0.001).

The analysis of autophagy related genes expression (Fig. 6) as well as conversion of LC3I to LC3II form (Supplementary Fig. 2) showed that GABA-containing and GABA-free supernatants significantly induced autophagy in stimulated MLNC, and GABA containing supernatants had the most prominent effects. The immunoregulatory effects of these supernatants correlated with the induction of autophagy, which is in accordance with many results pointing to a strong relation between autophagy related genes and immune regulation/homeostasis. In that sense, the beneficial role of ULK-1 protein kinase dependent autophagy has been proven for the infective and non-infective inflammatory injuries^58,59^. Similarly, the association between some inflammatory pathologies and deregulation of autophagy was been documented previously. Namely, Luo *et al*. showed that mRNA levels of *Becn1* were downregulated in patients with Systemic Lupus Erythematosus (SLE) and that the downregulation of *Becn1* significantly increased the incidence of nephritis^60^. Globular adiponectin, which possesses potent anti-inflammatory properties, inhibited LPS-stimulated inflammatory cytokines expression, at least in part, via p62 induction and autophagy activation in RAW2647 macrophages and mouse peritoneal macrophages^61^. Importantly, autophagy is actively implicated in the maintenance of stability and survival of regulatory T cells (Treg)^34^. Deletion of *ATG5* in Treg, leads to a loss of Treg and development of inflammatory disorders. The mice with loss of ATG5 showed a disrupted immune homeostasis of CD4^+^ and CD8^+^ cells that was associated with an increased IFN-γ expression.

As we have shown that GABA-containing and GABA-free supernatants induce autophagy, and that those containing GABA have more pronounced effects, we further tested if there are any differences between the effects of these supernatants on LC3 homolog, GABARAP. Interestingly, only the supernatants containing GABA (MRS/MSG and MRS/art. GABA) significantly stimulated the expression of *GABARAP* (Fig. 6g) in comparison to non-treated cells and the supernatants without GABA (MRS) (Fig. 6g). The exclusive stimulatory effects on GABARAP and stronger stimulatory effect of GABA containing supernatants on all other autophagy genes, correlated with the stronger immunoregulatory effects of these supernatants. These results suggested that GABA triggered signalling towards activation of GABARAP have an important role in autophagy-related immunoregulatory effects of *L. brevis* BGZLS10-17 supernatants. These results are in accordance with the findings of Salah *et al*. showing the potential role of GABARAP in tumorigeneses. Namely, these authors showed that the tumor-imposed suppression of immune response in tumor, such as the secretion of IL-1*β*, IL-6, IL-2 and IFN-*γ* by macrophages and lymphocytes^62^ depends on GABARAP activity. Additionally, these authors showed that the levels of TGF-*β*1 are significantly reduced in the serum of GABARAP KO mice.

### Immunoregulatory effects induced by GABA-contained in *Lactobacillus brevis* BGZLS10-17 supernatant depend on ATG5-mediated induction of autophagy

**Figure 7:**
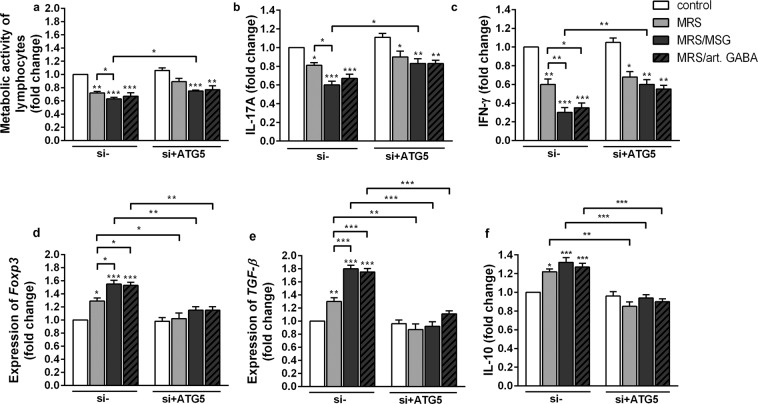
GABA-induced autophagy is involved in immune regulation. The effect of BGZLS 10-17 supernatants on the metabolic activity (**a**), production of IL-17A (**b**), production of IFN-γ (**c**), mRNA level of *Foxp3* (**d**), mRNA level of *TGF-β* (**e**), and production of IL-10 (**f**) of ConA stimulated MLNC transfected with either control siRNA or ATG5 siRNA, after 24 h treatment. MLNC were treated with 2.5% supernatant without GABA (MRS), 2.5% supernatant with 4 mM bacterial GABA (MRS/MSG), and 2.5% supernatant with addition of 4 mM artificial GABA (MRS/art. GABA). All values are presented as mean ± SD from three independent experiments. One-way ANOVA with the Dunnett’s test was used to compare multiple groups. The statistical significance is shown (*p < 0.05; **p < 0.01, ***p < 0.001).

In order to further investigate the role of *L. brevis* BGZLS10-17 supernatants-induced autophagy in its immunomodulatory effects, the autophagy was inhibited by the treatment of MLNC with *ATG5* gene silencing using siRNA technology. MLNC were treated with ATG5 siRNA or scrambled siRNA (control), and the effects on metabolic activity and immune functions were monitored after 24 h. It is important to notice that the immunoregulatory effects of all supernatants after 24 h (Figs. 4–7) were slightly weaker in comparison to the same treatment imposed for 72 h (Figs. 2 and 3c). The capacity of supernatants to induce the expression of *ATG5*, *p62* and *LC3*, were lowered significantly upon siRNA ATG5 treatment, compared to corresponding control (Supplementary Fig. 5a–c). The treatment of MLNC with siRNA specific for ATG5 (si + ATG5) completely silenced the supernatants-induced *ATG5* expression in MLNC after 24 h, so the levels of *ATG5* expression were similar in siRNA ATG5-treated controls and the supernatants-treated MLNC. At the other hand, the expression of *p62* and *LC3* were almost completely inhibited in siRNA ATG5 treated control MLNC. A significant, although not complete downregulation of supernatants-stimulated *p62* and *LC3* expression was observed in si + ATG5 MLNC compared to scramble siRNA-treated MLNC (Supplementary Fig. 5). However, significant differences between the effects of GABA-containing supernatants (MRS/art. GABA and MRS/MSG) and GABA-free (MRS) supernatant were not detected in MLNC treated with siRNA-ATG5. These results suggested that the GABA-containing supernatants of *L. brevis* BGZLS10-17 induced autophagy in MLNC via ATG5. However, the up-regulation of p62 and LC3-II expression in MLNC treated with si + ATG5 suggested that other soluble molecules produced by *L. brevis* BGZLS10-17 during 48 h cultivation induce autophagy in an ATG5-independent manner.

Interestingly, when autophagy in MLNC was inhibited by si + ATG5 (Fig. 7), the inhibitory effects of supernatants on metabolic activity and ConA-induced production of IFN-γ and IL-17 were significantly lower (Fig. 7a–c). Additionally, there were no more significant differences between the effects of GABA-containing and GABA-free supernatants on the production of IFN-γ and IL-17. However, si + ATG5 MLNC treated with supernatants still produced lower levels of these proinflammatory cytokines compared to si + ATG5 control MLNC, suggesting that the inhibition of these proinflammatory was completely ATG5 dependent, whereas the inhibition imposed by GABA-free supernatants was predominantly ATG5 independent. On the other hand, the inhibition of autophagy with si + ATG5 in MLNC completely abolished the stimulatory effects of the supernatants on the expression of Foxp3, *TGF-β* and IL-10 in MLNC (Fig. 7d–f). However, the GABA-free supernatants seem to inhibit the proinflammatory response of MLNC independent of GABA, ATG5 and down-stream FoxP3, IL-10 and TGF-β. The inhibitory potential of GABA-free supernatants could be mediated by direct actions on tolerogenic APC, since we found that OX62^+^ DC, B cells and Mf treated with this supernatant expressed lower levels of pro-inflammatory molecules (MHCII and CD80) and higher levels of immunoregulatory molecules (CTLA-4 and SIRP-α). These effects could be mediated by autophagy-dependent pathway, as we found a low induction of autophagy in these cells upon GABA-free supernatant treatment. These effects on APC could have been induced in autophagy independent way, as well, so further studies are needed to elucidate the mechanisms of action of GABA-free supernatants.

In conclusion, here we provided evidence that live-bacteria-cell-free GABA-containing and GABA-free supernatants produced by *L. brevis* BGZLS10-17 induces strong immunoregulatory effects on ConA-stimulated MLNC, and that GABA-containing supernatant have an additional immunoregulatory potential. Further, we showed that these additional effects of GABA are completely mediated by ATG5 dependent autophagy, but also that other soluble molecules produced by *L. brevis* BGZLS10-17 display immunomodulatory effects independent of ATG5.

## Materials and Methods

### Bacterial strain

*Lactobacillus brevis* BGZLS10-17, GABA-producing natural isolate from artisanal Zlatar cheese^22^ from the laboratory collection of the Laboratory for Molecular Microbiology, Institute of Molecular Genetics and Genetic Engineering (IMGGE), University of Belgrade, Serbia was used in this study. Strain *L. brevis* BGZLS10-17 was used to determine immunomodulatory effect, *in vitro*. The strain was grown 48 h at 37 °C anaerobically in de Man-Rogosa-Sharpe (MRS) broth (Merck, Darmstadt, Germany) and according to high-performance liquid chromatography (HPLC) analysis there was no GABA production^22^. To stimulate GABA production by *L. brevis* BGZLS10-17, MRS medium was supplemented with 0.6% of monosodium glutamate (MSG) (Acros organics, Morris Plains, NJ, United States) and the strain was cultivated likewise (this supernatant is annotated as MRS/MSG) as described in Sokovic Bajic *et al*.^22^. The concentration of GABA in MRS/MSG was measured by HPLC analysis. To investigate the role of GABA in MRS/MSG supernatant, supernatants from *L. brevis* BGZLS10-17 cultured during 48 h in MRS without MSG (MRS) and with addition of artificial GABA (Sigma) (MRS/art. GABA) in final concentration of 4 mM (the concentration of GABA in 2.5% MRS/MSG) were used as controls. All supernatants were filtered with 0.22 μm filters to be sure to have bacterial-cell free supernatants, neutralized (pH7) and kept at −20 °C.

### GABA production analysis

As described in Sokovic Bajic *et al*.^22^ the *L. brevis* BGZLS10-17 strain was incubated in MRS medium supplemented with 0.6% of MSG (Acros organics). The cells were harvested by centrifugation (4500 × g for 15 min at 4 °C) and 1 ml of the supernatant was evaporated up to 200 μl and diluted 2-fold by 7% acetic acid. The samples were then centrifuged at 8100 × g for 15 min at room temperature. The obtained supernatants were used for HPLC analysis^63^. The aliquots of 100 μl [bacterial supernatants and GABA standard (Sigma)] were filtrated through 0.22 μm filters and derivatized to phenylthiocarbamyl-GABA^64^. The derivatized samples were dissolved in 200 μl of the initial mobile phase, solution A (138 mM sodium acetate, pH 6.3, 6% acetonitrile, 0.05% triethylamine). HPLC separation was performed on the instrument of Thermo scientific 3000 equipped with a Hypersil gold column (Thermo Fisher Scientific, Waltham, MA, United States 150 × 4.6, 5 μm). The elution solvent system comprised of solution A, solution B (acetonitrile) and solution C (water). The elution program is shown in Supplementary Table 1. The amount of GABA production was calculated from the standard curve.

### Animals

Female dark agouty (DA) rats (inbred strain of rats, 8-10-week-old, weighing 200–250 g), used in the experiments were maintained in the animal facility of the Institute for biological research “Sinisa Stankovic”, Belgrade, Serbia. The rats were housed in the temperature-controlled room (22 ± 1 °C) with food and water available *ad libitum* under a 12 h light/dark cycle. Experiments were approved by the local ethics committee (Institute for Biological Research “Sinisa Stankovic”, No. 03-1/15). All experiments were performed in accordance with relevant guidelines and regulations.

### Isolation and treatment of mesenteric lymph node cells

Mesenteric lymph nodes (MLN) were isolated from DA rats and suspension of cells (MLNC) was prepared by mechanical disruption of MLN. The number of MLNC was determined by counting live cells after Tripan blue staining on light microscopy. MLNC (2.5 × 10^6^) were cultivated in 0.5 ml RPMI-1640 medium (Gibco™) supplemented with 10% FBS (Gibco™), 100 U/ml penicillin and 100 µg/ml streptomycin (Gibco™), in 24 well plate and were maintained during the treatment period at 37 °C in a humidified atmosphere containing 5% CO_2_. MLNC were stimulated with concanavalin A (ConA, Sigma-Aldrich, 2.5 µg/ml) and treated for 24 or 72 h with fresh MRS, GABA-free and GABA-containing *L. brevis* BGZLS10-17 supernatants in concentrations of 0.625%, 1.25%, 2.5%, 5%, and 10%. These concentrations of GABA-containing supernatants (MRS/MSG and MRS/art. GABA) contained 1 mM, 2 mM, 4 mM, 8 mM, and 16 mM GABA respectively, as determined by HPLC. To analyze the autophagic flux, the cells were exposed to lysosomotropic agent chloroquine (CQ) (Sigma-Aldrich) at a concentration of 25 μM simultaneously with treatments^19^. After 24 or 72 h of incubation, the cells and cell culture supernatants were collected and subjected to following analyses.

### Lactate dehydrogenase assay

The level of cytotoxicity in the cell cultures after 24 and 72 h was measured by lactate dehydrogenase (LDH) Cytotoxicity Assay Kit (Thermo Fisher Scientific) which detects LDH released from dead cells, as described in Sokovic Bajic *et al*.^22^. After treatments, supernatants were collected and LDH activity was determined by following the manufacturer’s instructions. The absorbance was measured at 450 nm on a microplate reader (Tecan Austria GmbH, Grödig, Austria).

### Measurement of metabolic activity of mesenteric lymph node cells

As described previously by Mosmann^65^, the effect of *L. brevis* BGZLS10-17 SN on metabolic activity of MLNC cultured for 24–72 h was examined by MTT assay [3-(4, 5-dimethylthiazol-2-yl)-2, 5-diphenyl tetrazolium bromide] (Serva, Electrophoresis GmbH, Heidelberg, Germany). After the treatment, the MTT dissolved in Phenol Red-Free RPMI media was added at the final concentration of 0.5 mg/ml. After 4 h of incubation, at 37 °C with 5% CO2 10% SDS-0.01 N HCl was added to dissolve formazan. The absorbance was measured with a microplate reader (Tecan) at a wavelength of 570 nm with references wavelength at 640 nm. The results are presented as the percentage of metabolic activity of treated cells compared to the control (100%).

### Measurement of proliferation of mesenteric lymph node cells

In order to analyse the effect of *L. brevis* BGZLS10-17 supernatant on polyclonal proliferation of MLNC, the cells were labeled with carboxyfluorescein succinimidyl ester (CFSE, 1 µM, Thermo Fisher Scientific, Waltham, MA, USA) according to manufacturer’s protocol, and then treated with Concanavalin A (2.5ug/ml, Sigma) either in the presence or absence of *L. brevis* BGZLS10-17 supernatants. The proliferation of CFSE-labeled MLNC was analyzed within live cells, as determined by simultaneous staining with propidium iodide (PI, Sigma, 10ug/ml) after the cultures, by flow cytometry according to Tomić *et al*.^66^. The percentage of proliferation was calculated using the proliferation fit statistics in FCS Express 4 software (De Novo Software, Glendale, CA, USA). The relative proliferation in suppression assays was calculated as the percentage of proliferation relative to control (100%).

### Quantification of cytokines

Cytokines concentration in 24 and 72 h cell culture supernatants was determined by enzyme linked immunosorbent assay (ELISA). For interleukin (IL)-10 and interferon (IFN)-γ detection, specific DuoSet ELISA were used according to the manufacturer’s instructions (R&D Systems, Minneapolis, MN, USA). For IL-17A detection, rat IL-17A (homodimer) ELISA Ready-SET-Go was used according to the manufacturer’s instructions (eBioscience, San Diego, CA, USA). Samples were analyzed in duplicates and the results were calculated using the standard curves.

### Quantitative real-time PCR

To analyze the level of mRNA for different functional molecules total RNA was extracted from MLNC collected from 24 h cultures as previously described in Sokovic Bajic *et al*.^22^. Denaturing solution (4 M guanidine thiocyanate, 25 mM sodium citrate, 0.1 M b-mercaptoethanol, 0.5% [wt/vol] N-lauroylsarcosinate sodium salt) was used for cell lysis followed by acid phenol (pH 4) extractions and isopropanol precipitation. 200 ng of total RNA was used for generation of cDNA according to the reverse transcriptase manufacturer’s protocol (Thermo Scientific). Quantitative PCR was realized in 7500 real-time PCR system (Applied Biosystems, Waltham, MA, USA) using KAPA SYBR Fast qPCR Kit (Kapa Biosystems,Wilmington, MA, USA) under the following conditions: 3 min at 95 °C activation, 40 cycles of 15 sec at 95 °C and 60 sec at 60 °C. The primers used (Table 1) were purchased from Thermo Fisher Scientific.

**Table 1:** List of primers used in this study.

Primer name	Primer sequence 5′-3′	Reference
β-actin forward β-actin reverse	AGCCATGTACGTAGCCATCC CTCTCAGCTGTGGTGGTGAA	^67^
TGF-β forward TGF-β reverse	GCTGAACCAAGGAGACGGAATA ACCTCGACGTTTGGGACTGA	This work
FoxP3 forward FoxP3 reverse	CCCAGGAAAGACAGCAACCTT CTGCTTGGCAGTGCTTGAGAA	^68^
ULK 1 forward ULK1 reverse	CTCCCCAAGTGGGAACCATC GGGACGAACGACATGGAAGT	This work
UVRAG forward UVRAG reverse	CTGTACACCTGACTCCCACG GGGGCTCTCCTGTTACAAGT	This work
Becn1 forward Becn 1 reverse	CAATACCAGAATCCACAAAAGC AGGGAAGAGGGAAAGGACAG	Personal communication
ATG 5 forward ATG 5 reverse	CGGTGCAAGGATGCAGTTGAG TTCTGCAGTCCCATCCAGAG	This work
LC3 forward LC3 reverse	GACTTCCGGAAAGCTCTGCT ACCAGCATCGTAGAGGGTCT	^69^
GABARAP forward GABARAP reverse	AAAGCTCGGATAGGGGACCT CACTGGTGGGTCGAATGACA	This work
p62 forward P62 reverse	TCCCTGTCAAGCAGTATCC TCCTCCTTGGCTTTGTCTC	^69^

### Western blot

Proteins for detection of LC3I/LC3II level were isolated from MLNC collected from 24 h cultures using radioimmunoprecipitation assay (RIPA) buffer and subsequently subjected to Western blot analysis as described by Dinić *et al*.^19^. Briefly, the extracted proteins (10 μg) were separated on 12% SDS–PAGE and transferred to 0.2 mm nitrocellulose membrane (GE Healthcare, Chicago, IL, United States) using Bio-Rad Mini trans-blot system (Bio-Rad, Hercules, CA, United States). The membranes were incubated overnight at 4 °C with anti-LC3 (1:2000; Thermo Fischer Scientific) and anti-β-actin (1:1000; Thermo Fisher Scientific). The membranes were washed and incubated with appropriate HPR-conjugated secondary antibodies (goat anti-rabbit; 1:10000; Thermo Fisher Scientific) for 1 h at room temperature. Proteins were detected by enhanced chemiluminescence (Immobilon Western, Merck Millipore). The intensity of the bands was quantified using ImageJ software as described previously in Sokovic Bajic *et al*.^22^.

### RNA interference

MLNC were washed twice in OptiMEM medium (Thermo Fischer Scientific, Waltham, MA), and seeded in 24-well plates (1 × 10^6^ cells/well) in OptiMEM medium. MLNC were then transfected with scrambled small interfering RNA (siRNA) control, or siRNA targeting rat ATG5 (Ambion, Applied Biosystems), using Lipofectamine 2000 Transfection Reagent (Thermo Fischer Scientific, Waltham, MA) according to the manufacturer’s instructions. After 8 h, an equal volume of complete RPMI medium was added for the next 16 h, and the transfected MLNC were treated with *L. brevis* BGZLS10-17 supernatants during next 24 h and used in assays according to Tomić *et al*.^47^.

### Flow cytometry

The effects of *L. brevis* BGZLS10-17 supernatants on LC3 flux within MLNC was analyzed in MLNC collected after 72 h cultures pre-treated with CQ, by flow cytometry. The expression of functional molecules on selected immune cells was carried out on similar cultures that were not treated with CQ. After the cultures, MLNC were washed twice in phosphate buffer saline (PBS) containing 0.1% NaN3 and 2% FCS and surface stained with the following, directly conjugated mouse-anti-rat monoclonal antibodies (mAbs): CD3 Alexa Fluor 647 (clone: 1F4), CD11b Alexa Fluor 647 (OX-42), CD4 phycoerythrin (PE) (W3/25), MHC class II RT1B (OX-6) PE, CD68 (ED1) Alexa 647, isotype control rat IgG biotin, isotype control IgG Alexa 647 (Bio-Rad), CD161 biotin (10/78), alpha E2 integrin (OX-62) biotin (R&D Systems), CD80 PE (3H5), CD45R B220 FITC (HIS 24), isotype control rat IgG pe (BD Biosciences), CD152 CTLA-4 (14D3) biotin, CD172a SIRP-α (P84) biotin, isotype control Rat IgG FITC (ThermoFisher Scientific). After the staining with biotin labeled mAbs, the cells were washed and incubated for 15 minutes with streptavidin PEcy7 (Biolegend) and then washed with PBS/NaN3. The surface staining was followed by fixation and permeabilization using BD fixation/permeabilization kit, according to manufacturer’s protocol. Intracellular staining of LC3 was carried out in permeabilization buffer using rabbit anti-rat LC3 Ab, followed by goat-anti-rabbit IgG Alexa 647 or goat-anti-rabbit IgG Alexa 488 (ThermoFisher Scientific). Due to permeabilization, this method preferentially detects membrane bond LC3II, over cytosolic LC3I^31^ and the sensitivity is increased by labeling with secondary Abs. The cells were gated according to their size and granularity upon exclusion of doublets, and the compensation overflow was determined using single stained samples. Non-specific fluorescence was detected according to isotype control mAbs, fluorescence minus one (FMO) controls, or in the case of LC3 detection, omission of the primary Ab.

### Statistical analysis

All data, except graphs presenting FACS analysis, are presented as mean values ± standard error of the mean. One-way ANOVA with the Dunnett’s test was used to compare multiple groups. The differences between control and experimental groups were compared using Student’s t-test. Values at p < 0.05 or less were considered statistically significant. All experiments were repeated at least three times. The graphs presenting FACS analysis are representatives from two experiments. Statistical analysis and graph design were carried out using GraphPad Prism Software.

### Ethical approval

Experiments were approved by the local ethics committee (Institute for Biological Research “Sinisa Stankovic”, No. 03-1/15). All experiments were performed in accordance with relevant guidelines and regulations.

## Supplementary information

Supplementary data.
