# Supplementary material for: GABA potentiate the immunoregulatory effects of Lactobacillus brevis BGZLS10-17 via ATG5-dependent autophagy in vitro
Source: Sci Rep. 2020 Jan 28;10:1347. doi: 10.1038/s41598-020-58177-2 (PMC6987229; doi:10.1038/s41598-020-58177-2)
Supplement: Supplementary file 1 — Supplementary data. [file 41598_2020_58177_MOESM1_ESM.docx]

GABA potentiate the immunoregulatory effects of *Lactobacillus brevis* BGZLS10-17 via ATG5-dependent autophagy *in vitro*

Svetlana Soković Bajić^1^, Jelena Đokić^1^*, Miroslav Dinić^1^, Sergej Tomić^2^, Nikola Popović^1^, Emilija Brdarić^1^, Nataša Golić^1^, Maja Tolinački^1^

Supplementary table 1. Elution program of HPLC

| Time (min) | Solution A (%) | Solution B (%) | Solution C (%) | Flow (ml/min) |
| --- | --- | --- | --- | --- |
| 0 | 60 | 12 | 28 | 0.6 |
| 6 | 60 | 12 | 28 | 0.6 |
| 6.1 | 20 | 13.5 | 66.5 | 0.4 |
| 22 | 20 | 13.5 | 66.5 | 0.4 |
| 25 | 60 | 12 | 28 | 0.6 |
| 40 | 60 | 12 | 28 | 0.6 |


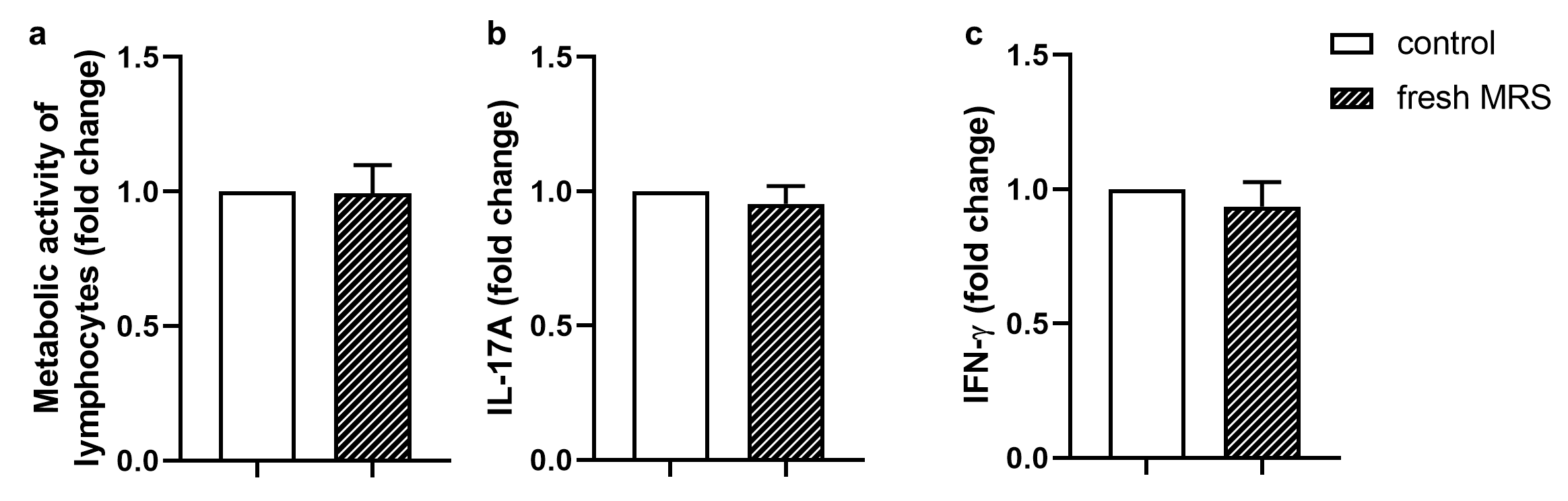


**Supplementary figure 1.** The effect of fresh MRS. The effect of fresh MRS (2.5%) on metabolic activity (a), and production of proinflammatory cytokines IL-17A (b) and IFN-γ (c) by ConA stimulated MLNC, after the 72 h treatment.





**Supplementary figure 2.** The influence of BGZLS10-17 supernatants on autophagy of ConA stimulated MLNC. Western blot and densitometric analysis (a) of LC3 conversion in MLNC. MLNC were treated with 2.5% supernatant without GABA (MRS), 2.5% supernatant with 4 mM bacterial GABA (MRS/MSG), and 2.5% supernatant with addition of 4 mM artificial GABA (MRS/art. GABA) for 24 h. All values are presented as mean ± SD from three independent experiments. One-way ANOVA with the Tukey’s post hoc test was used to compare multiple groups. The statistical significance is shown (*p < 0.05, ***p < 0.001). Autophagy flux assessment on ConA stimulated MLNC (b) representative WB and densitometric analysis of LC3 conversion in MLNC treated for 24 h with 25 μM CQ and supernatant without GABA (MRS), supernatant with 4 mM bacterial GABA (MRS/MSG), and supernatant with addition of 4 mM artificial GABA (MRS/art. GABA).





**Supplementary figure 3.** The influence of BGZLS10-17 supernatants on the expression of pro-inflammatory molecules CD80 and MHC-II, immunosuppressive molecule CTLA-4, and autophagy of ConA stimulated B cells. The percentage and mean of HIS24^+^CD80^+^, HIS24^+^CTLA-4^+^, HIS24^+^MHC-II^+^, HIS24^+^LC3^+^ were assessed by flow cytometry. MLNC were treated with 2.5% supernatant without GABA (MRS), 2.5% supernatant with 4 mM bacterial GABA (MRS/MSG), and 2.5% supernatant with addition of 4 mM artificial GABA (MRS/art. GABA) for 24 h. Representative histograms from two experiments are presented.





**Supplementary figure 4.** The influence of BGZLS10-17’s supernatants on the expression of pro-inflammatory molecule MHC-II, immunosuppressive molecule SIRP-α, and autophagy of macrophages. The percentage and mean of CD68^+^MHC-II^+^, CD80^+^SIRPα^+^, and CD80^+^LC3^+^ were assessed by flow cytometry. MLNC were treated with 2.5% supernatant without GABA (MRS), 2.5% supernatant with 4 mM bacterial GABA (MRS/MSG), and 2.5% supernatant with addition of 4 mM artificial GABA (MRS/art. GABA) for 24 hours. Representative histograms from two experiments are presented.


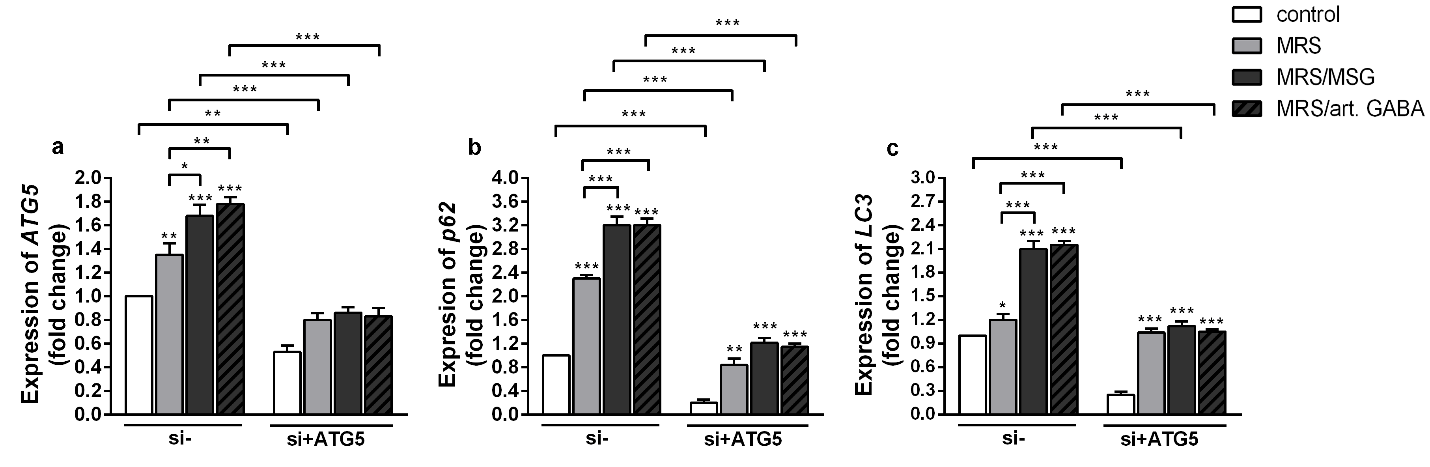


**Supplementary figure 5.** Silencing the autophagy with si+ATG 5. The effect of BGZLS10-17’s supernatants on mRNA levels of *ATG5* (a), *p62* (b), and *LC3* (c) of ConA stimulated MLNC transfected with either control siRNA or ATG5 siRNA, after 24 hours treatment. MLNC were treated with 2.5% supernatant without GABA (MRS), 2.5% supernatant with 4 mM bacterial GABA (MRS/MSG), and 2.5% supernatant with addition of 4 mM artificial GABA (MRS/art. GABA). All values are presented as mean ± SD from three independent experiments. One-way ANOVA with the Tukey’s post hoc test was used to compare multiple groups. The statistical significance is shown (*p < 0.05; **p < 0.01, ***p < 0.001).
